# A novel gene delivery composite system based on biodegradable folate-poly (ester amine) polymer and thermosensitive hydrogel for sustained gene release

**DOI:** 10.1038/srep21402

**Published:** 2016-02-17

**Authors:** Yi Yang, Hang Zhao, YanPeng Jia, QingFa Guo, Ying Qu, Jing Su, XiaoLing Lu, YongXiang Zhao, ZhiYong Qian

**Affiliations:** 1 State Key Laboratory of Biotherapy and Cancer Center, West China Hospital, Sichuan University, and Collaborative Innovation Center for Biotherapy, Chengdu, 610041, P.R. China; 2 National Center for International Research of Biological Targeting Diagnosis and Therapy, Guangxi Key Laboratory of Biological Targeting Diagnosis and Therapy Research, Collaborative Innovation Center for Targeting Tumor Diagnosis and Therapy, Guangxi Medical University, 22 Shuang Yong Rd. Nanning, Guangxi 530021, China

## Abstract

Local anti-oncogene delivery providing high local concentration of gene, increasing antitumor effect and decreasing systemic side effects is currently attracting interest in cancer therapy. In this paper, a novel local sustained anti-oncogene delivery system, PECE thermoresponsive hydrogel containing folate-poly (ester amine) (FA-PEA) polymer/DNA (tumor suppressor) complexes, is demonstrated. First, a tumor-targeted biodegradable folate-poly (ester amine) (FA-PEA) polymer based on low-molecular-weight polyethyleneimine (PEI) was synthesized and characterized, and the application for targeted gene delivery was investigated. The polymer had slight cytotoxicity and high transfection efficiency *in vitro* compared with PEI 25k, which indicated that FA-PEA was a potential vector for targeted gene delivery. Meanwhile, we successfully prepared a thermoresponsive PECE hydrogel composite containing FA-PEA/DNA complexes which could contain the genes and slowly release the genes into cells. We concluded the folate-poly (ester amine) (FA-PEA) polymer would be useful for targeted gene delivery, and the novel gene delivery composite based on biodegradable folate-poly (ester amine) polymer and thermosensitive PECE hydrogel showed potential for sustained gene release.

Gene therapy, which means the genetic modification of pathological cells through the delivery of DNA or RNA that can up- or down-regulate genes, holds great promise for treating diseases ranging from inherited disorders to acquired cancers^1^,^2^,^3^. Genes can be delivered into cells with the use of modified viruses or non-viral gene delivery vectors. Though viruses have the inherent function of cell invasion that is followed by the expression of genes into the host cell, the use of viruses in gene delivery is limited by the side reaction such as the host’s immune response, insertional mutagenesis leading to death and so on^4^. Among non-viral gene delivery vectors, polyethylenimine (PEI) derivatives have been commonly used in gene delivery for their high transfection efficiency, biodegradability and low cytotoxicity^5^,^6^. Poly (ester amine) (PEA), based on L-lactide and low molecular weight polyethylenimine, has been widely used in gene delivery for cancer therapy because of high transfection efficiency, favorable biodegradability, preferable biocompatibility and low cytotoxicity^5^,^7^,^8^.

In spite of the great promise of biodegradable polymer-mediated gene delivery for cancer therapy, the broad clinical application still faces great challenges, including targeted gene delivery, sustained release and local gene delivery and so on^9^,^10^,^11^. A large number of new approaches have been reported that have overcome some shortcomings of polymer carriers in gene delivery. For example, tumor-specific peptide and antibodies were linked to cationic polymers, and these new vectors enabled certain degrees of success^12^,^13^,^14^,^15^. Besides, controlled release of genes from biodegradable polymeric microspheres using PLGA^16^,^17^ was suggested as a potential vector for continuous gene release in a desired local tissue. However, the degradation and structural change of genes in PLGA microspheres were inevitable when preparing the gene-loaded microspheres^18^,^19^. The study of local delivery of genes through a hydrogel matrix has attracted much attention^3^,^4^,^20^,^21^,^22^. Raw DNA encapsulated in collagen hydrogels was able to promote bone regeneration *in vivo*, but gene transfection efficiency was very low^23^,^24^. Based on this, researchers used cationic polymers to condense the genes into particles, and dispersed the complexes into hydrogel, the prepared hydrogel composite system could both retain the DNA in the matrix and significantly increase the transfection efficiency^3^.

Based on the above consideration, we developed a multifunctional thermoresponsive hydrogel gene delivery system, based on thermoresponsive hydrogel PECE (PEG_550_-PCL_2200_-PEG_550_) for retaining genes in the matrix and biodegradable folate-poly (ester amine) polymer for gene delivery into cancer cells, which met the demands of tumor-targeted gene delivery, safety, sustained gene release and local gene delivery. Therefore, in this study, we synthesized a biodegradable poly (ester amine) polymer modified by folic acid (FA-PEA) which had been widely used in targeted delivery of genes because of the folate receptor’s over-expression in many tumors^25^,^26^,^27^,^28^. Afterwards, the FA-PEA polymer was used to condense the gene into particles, and the gene complexes were dispersed into a thermosensitive hydrogel PECE which had excellent sol-gel phase transition behavior and sustained release of drug reported by our group^29^,^30^,^31^. The prepared hydrogel system could slowly release genes into local tissue and selectively deliver genes into cancer cells rather than normal cells.

## Results

### Synthesis and characterization of PEA and FA-PEA

**Figure 1:**
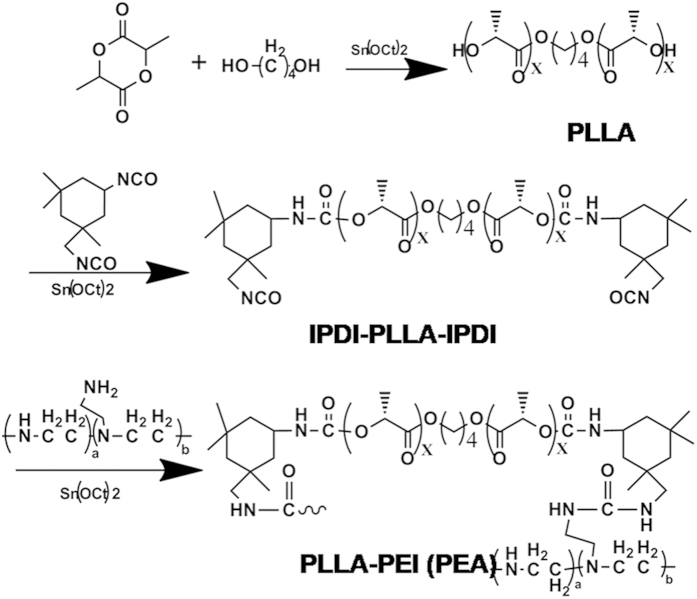
Synthetic route of PLLA-PEI (PEA) polymer. The Fig. was drawn with ChemBioDraw Ultra 12.0 by the author Y. Y.

**Figure 2:**
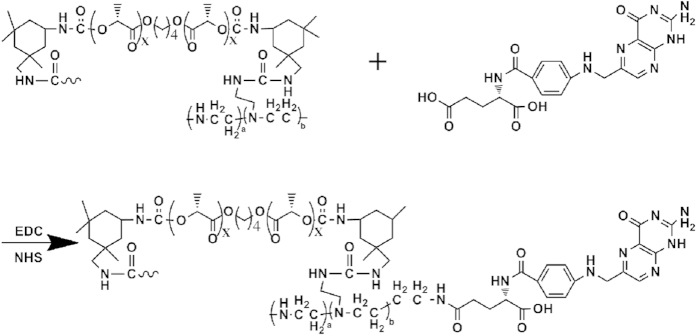
Synthetic route of FA-PEA polymer. The Fig. was drawn with ChemBioDraw Ultra 12.0 by the author Y. Y.

The poly (ester amine) (PEAs) polymer was synthesized in three steps according to Fig. 1. First, we obtained poly (L-lactide) (PLLA) by ring-opening polymerization of L-lactide initiated by 1,4-butanediol, then NCO-ended PLLA polymer was obtained according to the reaction of isophorone di-isocyanate (IPDI) and PLLA, finally we got poly (ester amine) (PEA) polymer by the reaction of NCO- on IPDI-PLLA-IPDI and NH_2_ on low molecular weight branched PEI 1800. The FA-PEA polymer was obtained by the reaction of carboxyl on FA and amidogen on PEA, as shown in Fig. 2.

**Figure 3:**
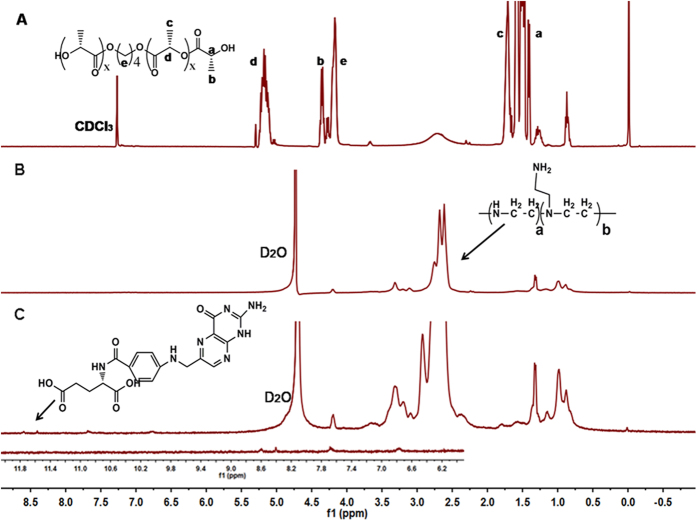
^1^H-NMR spectra of the synthesized polymers. (**A**) PLLA in CDCl3, (**B**) PLLA-PEI and (**C**) FA-PEA in D2O, the^1^H-NMR spectra of these three polymers was merged with Adobe Photoshop.

Figure 3 shows the ^1^H-NMR spectrum of the synthesized PLLA macromer, PEA and FA-PEA. In Fig. 3A, peaks “c” and “a” were attributed to the methyl protons of -O-CH(CH_3_)CO- and HO-CH(CH_3_)-CO- in PLLA, “e” was the peak of methylene protons of the –CH_2_- in 1,4-butanediol, “d” and “b” were assigned to the tertiary protons of –O-CH(CH_3_)CO- and HO-CH(CH_3_)CO- in PLLA, these peaks indicated the success of PLLA synthesis, and the molecular weight of PLLA was about 454Da analyzed by gel permeation chromatography (GPC). The ^1^H-NMR spectrum of PLLA-PEI polymer is shown in Fig. 3B, the broad peaks between 2ppm and 3ppm were attributed to the protons of –NHCH_2_CH_2_- in the PEI moiety of PLLA-PEI, the characteristic peaks of PLLA were barely discovered because of the lower molecular weight of PLLA compared with PEI. The ^1^H-NMR spectrum of the resulting FA-PEA based on the reaction of FA and PEA is shown in Fig. 3C, the small, broad peaks were the characteristic peaks of folic acid (FA), the peaks around 10ppm on behalf of the carboxyl did not appear in this Fig., which also meant the successful synthesis of FA-PEA.

### *In vitro* cytotoxicity

**Figure 4:**
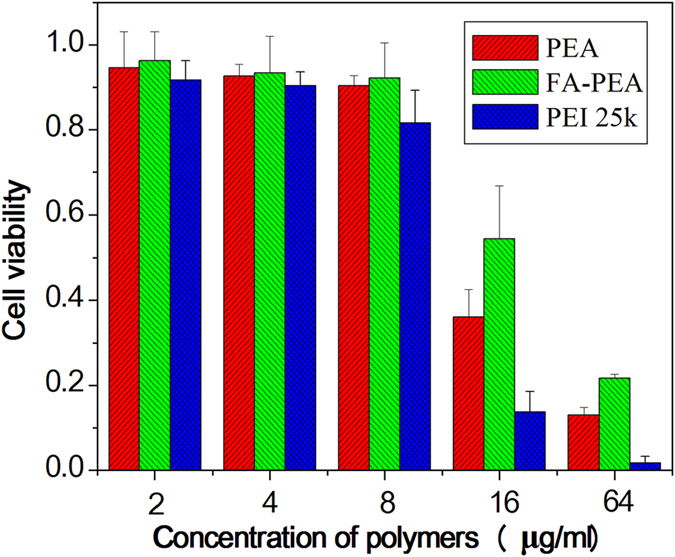
Cytotoxicity of PEA, FA-PEA and PEI 25k in C26 cell lines. Cell viabilities after treatment of polymers at various concentrations were determined by MTT assay. The results were presented as mean ± standard deviation (n = 6).

The cytotoxicity of the PEA and FA-PEA polymers *in vitro* compared with PEI 25k was evaluated by MTT assay and the results are shown in Fig. 4, The Cell Lines used in this study was C26. Cell viability was calculated according to the following equation: Cell viability (%) = (OD_sample_−OD_blank_/OD_control_−OD_blank_), where OD_sample_ was the absorbance of the solution of the cells cultured with the polymers and PEI 25k; OD_blank_ was the absorbance of the medium; and OD_control_ was the absorbance of the solution of the cells cultured with the medium only. Both PEI derivants all showed dose-dependent effect on cytotoxicity, and showed little cytotoxicity at the low concentration (≤8μg/ml). We also found that the cytotoxicity of PEA modified by folic acid (FA) was clearly lower than that of PEA at almost all the concentrations, both PEA and FA-PEA were all found to have significant cytotoxicity when the concentration was higher than 16μg/ml. However, we could clearly see that the PEI derivatives had lower cytotoxicity compared with PEI 25 k, which might be caused by PLLA that decreased the positive charges of PEI.

### Gel retardation assay

**Figure 5:**
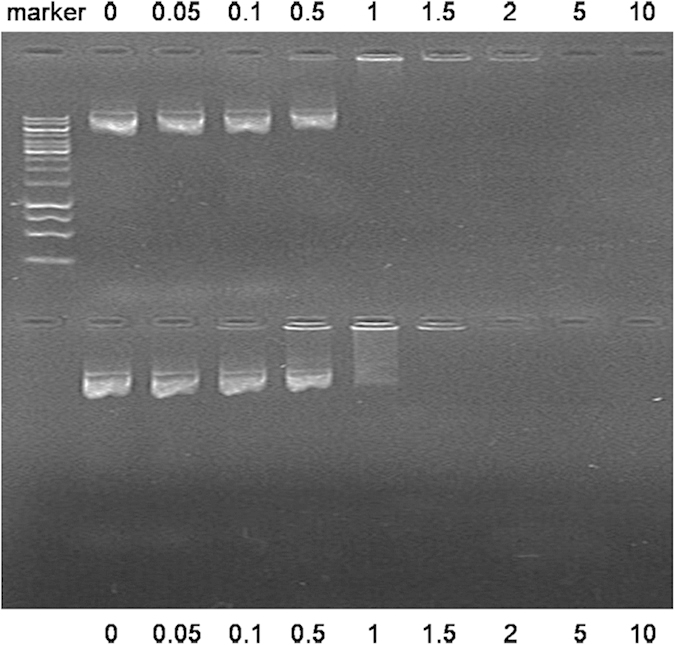
Agarose gel electrophoresis of polymer/gene complexes. PEA/DNA complexes (up) and FA-PEA/DNA complexes (down) were formed at various ratios using 1 × TAE buffer (constant voltage of 110 V, 20 min) and stained with SYBR gold (1×). Lane codes: described as shown in fugure.

In this study, agarose gel electrophoresis was carried out to determine the optimal concentration for complete condensation of DNA. Figure 5 shows the results of agarose gel electrophoresis of the polymers/DNA complexes at various ratios (from 0.05 to 10), and raw DNA was used as the control group. It was observed that with the increase in the ratio of the polymers, there was a reasonable decrease in the electrophoretic mobility. The PEA/DNA complexes showed complete retardation and the band disappeared completely when the weight ratio (PEA to gene) was 1. Meanwhile, a similar result was obtained for FA-PEA/DNA complex when the ratio was 1.5, this phenomenon might be caused by the less PEI content of FA-PEA compared with PEA.

### Sol-gel transition behavior of hydrogel

**Figure 6:**
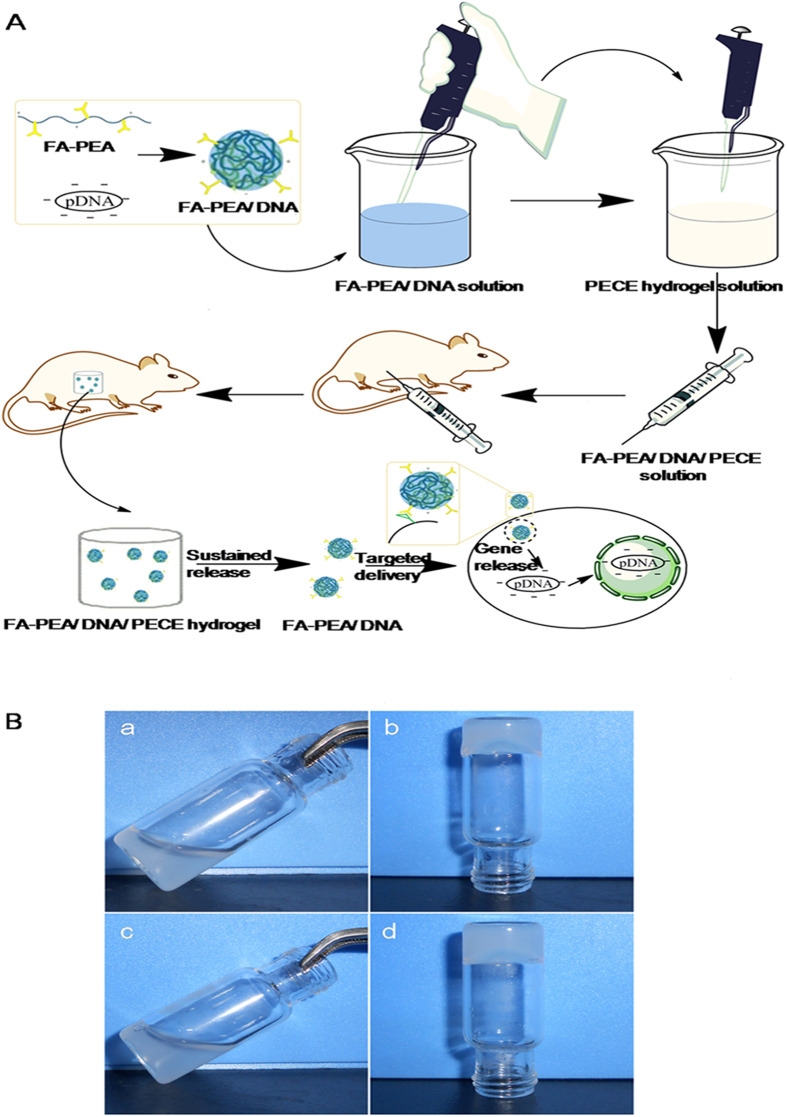
Formation of hydrogel loading gene. Preparation and intracorporal process of hydrogel (**A**); sol-gel transition behavior at room temperature and at 37 °C **(B)**, hydrogel (20% weight ratio of PECE) at room temperature (a) and at 37 °C (b), hydrogel (loading FA-PEA/DNA, the weight ratio of PECE was 20%) at room temperature (c) and at 37 °C (d). All parts of Fig. 6A were drawn with ChmoBioDraw Ultra by the author Y. Y.

Figure 6A shows the preparation and intracorporal process of hydrogel loading genes. FA-PEA/DNA complexes were prepared and added into thermosensitive PECE hydrogel solution, which was intraperitoneally injected into tumor-bearing mice afterwards, the sol-state hydrogel immediately changed into gel-state and kept in the abdominal cavity of the mice. With the degradation of PECE hydrogel, the FA-PEA/DNA complexes were slowly released and targeted into certain tumor cells, such as colorectal cancer cells, prostate cancer cells and so on^25^,^26^,^27^,^28^ which had over-expressed folate receptors. Finally, the antitumor genes were released with the disaggregation of FA-PEA/DNA complexes, and played a role in tumor inhibition.

As shown in Fig. 6B both PECE hydrogel and FA-PEA/DNA/PECE hydrogel were sol state at room temperature, and transformed into gel state at 37 °C. The sol-gel transition phase behavior indicated that the addition of FA-PEA/DNA complexes had no effect on the sol-gel transition behavior of the thermosensitive PECE hydrogel.

Besides, the rheologies of PECE hydrogel and FA-PEA/DNA/PECE hydrogel were also analyzed by ARE2000ex rheometer (TA Instruments, USA) according to tube-inverting method. The lower critical gelation temperatures of PECE hydrogel and FA-PEA/DNA/PECE hydrogel were 36.17 °C and 36.21 °C, and the upper critical gelation temperature of PECE hydrogel and FA-PEA/DNA/PECE hydrogel were 50.36 °C and 49.85 °C, respectively. So the adding of FA-PEA/DNA complexes had little or no effect on sol-gel transition behavior of PECE hydrogel.

### *In vitro* transfection

**Figure 7:**
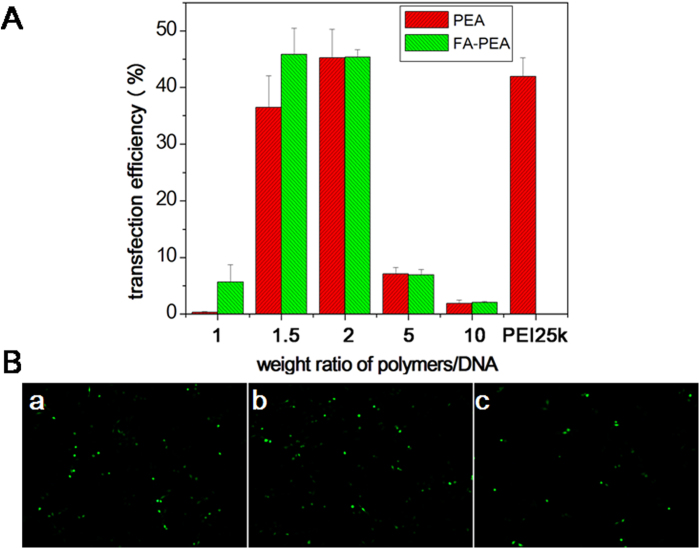
Transfection *in vitro*. Transfection efficiency of eGFP in C26 cell lines analyzed by (**A**) flow cytometric analysis, the results were presented as mean ± standard deviation (n = 3); Fluorescence inversion microscopy images of the C26 cell lines (**B**) by the eGFP polyplexes with PEA (**B**-a), FA-PEA (**B**-b) and PEI 25k (**B**-c), ×100.

The gene could be delivered into cells with the help of PEA and FA-PEA polymers. To test this hypothesis, eGFP plasmid was delivered into C26 cell lines mediated by PEA and FA-PEA polymers. Fluorescence inversion microscopy was employed to investigate their transfection efficiency qualitatively and the results are presented in Fig. 7B. It was found that with the help of these polymers, gene was delivered into the cells successfully.

Flow cytometry was further applied to quantitative study of their transfection efficiency and the results are presented in Fig. 7A. The transfection efficeiency in C26 cells presented a peak pattern with the increase in the ratio of polymers/DNA, which might be caused by the cytotoxicity of the polymers. The maximum transfection efficiency of PEA/DNA, FA-PEA/DNA and PEI/DNA complexes was nearly equal in the C26 cell when the ratio of polymer/DNA was 2. Based on the above results, the optimum weight ratio of polymer to DNA was determined as 2, and the weight ratio of polymer to DNA in all of the following experiments was 2 without further statement.

### Apoptosis *in vitro*

The apoptosis of colon cancer cells *in vitro* induced by the treatments of PEA/WIF-1 and FA-PEA/WIF-1 complexes was qualitatively detected by Hoechst 33258/PI and DAPI respectively.

**Figure 8:**
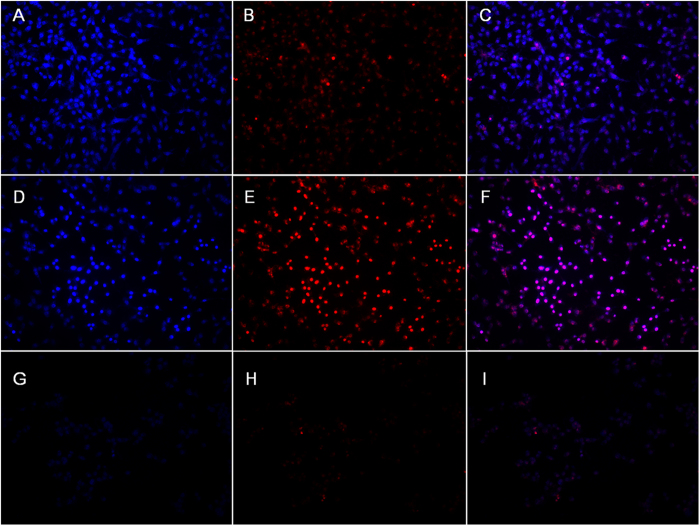
Apoptosis *in vitro*. The C26 cells were dyed with Hoechst 33258/PI after being treated with PEA/WIF-1 (**A–C**), FA-PEA/WIF-1 (**D–F**), free WIF-1 (**G–I**). The apoptotic cell was stained by Hoechst 33258 and revealed by blue in fluorescence inversion microscopy images (**A,D,G**); the apoptotic necrotic cell was stained by PI and presented by red(**B,E,H**); the blue and red fields of fluorescence inversion microscopy images of each sample were merged (**C,F,I**), ×200.

The nuclei of cells were stained by Hoechst 33258 and appeared blue in fluorescence inversion microscopy, but the dye could be excluded from viable cells; Necrotic and late apoptotic cells were stained by PI and showed red fluorescence^32^. Therefore, we could detect normal cells (weak red and weak blue fluorescence), apoptosis cells (blue and weak red fluorescence) and necrotic cells (red and blue fluorescence). As shown in Fig. 8, WIF-1 had no effect on C26 cell lines, while apoptotic and necrotic cells were observed when the cells were treated with PEA/WIF-1 or FA-PEA/WIF-1 complexes. These results indicated that with the help of PEA and FA-PEA, WIF-1 was successful delivered and expressed in C26 cells, and it also revealed the excellent antitumor effect of WIF-1.

**Figure 9:**
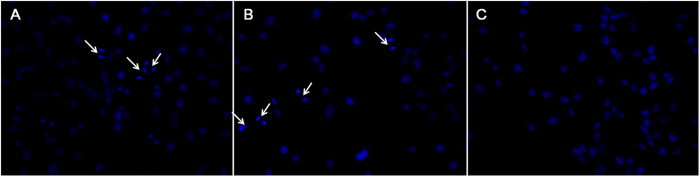
Apoptosis body of colorectal cancer cell C26. The cellular nucleus was dyed with DAPI after being incubated with PEA/WIF-1 (**A**), FA-PEA/WIF-1 (**B**), free WIF-1 (**C**). The arrows in the fluorescence inversion microscopy images were pointing to the apoptosis body, ×400.

DAPI is a fluorescent dye that could strongly combine with nuclear genes, therefore nuclear changes associated with apoptosis^33^ could be monitored and the results are shown in Fig. 9. We could recognize the apoptotic cells with chromatin condensation and nuclear fragmentation (blue, in crescent shape or with apoptotic bodies) and normal cells (weak blue, without chromatin condensation or nuclear fragmentation). There was apoptosis when the cells were treated with PEA/WIF-1 complexes or FA-PEA/WIF-1 complexes, and the results are in good agreement with that of Hoechst 33258/PI staining.

**Figure 10:**
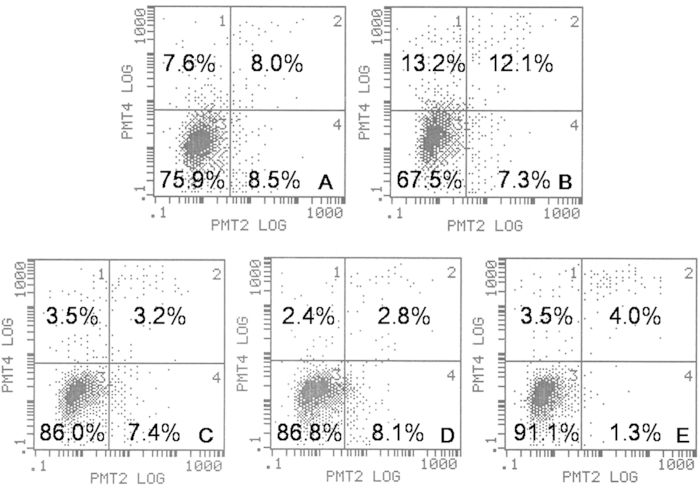
Apoptosis *in vitro* determined by flow cytometric analysis with Annexin-V/PI staining. The C26 cells were treated with PEA/WIF-1 (**A**), FA-PEA/WIF-1 (**B**), PEA (**C**), FA-PEA (**D**), free WIF-1 (**E**). The lower-left (3), upper-left (2), upper-right (4) and lower-right (1) quadrants in each panel represent the populations of normal, early and late apoptotic, and apoptotic necrotic cells, respectively.

Besides, the apoptosis was determined quantitatively by flow cytometry when stained with an Annexin V-FITC Apoptosis Detection Kit. Figure 10 shows that the apoptotic rate of colon cells were 16.5%, 19.4%, 10.6%, 10.9% and 5.3% after incubation with PEA/WIF-1, FA-PEA/WIF-1, PEA, FA-PEA and WIF-1 plasmid respectively, which was also in accordance with the results of Hoechst 33258/PI and DAPI staining.

### Sustained release of the gene from PECE hydrogel

**Figure 11:**
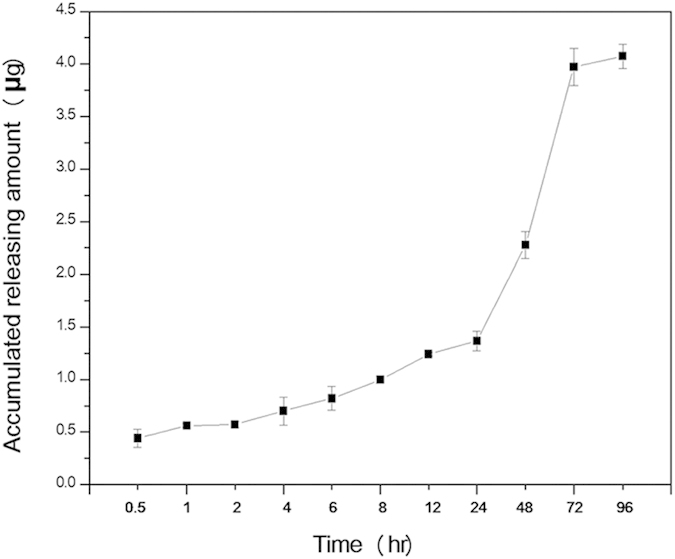
Release of gene from PECE hydrogel. The weight ratio of PECE was 20%; the initial concentration of gene was 20 μg), and the results were presented as mean ± SD (n = 3).

As shown in Fig. 11, more than 5% DNA (about 1.24 μg) was released from FA-PEA/DNA encapsulated PECE hydrogel in PBS 7.4 over the first 24 hours, following a more rapid release in the next 2 days, which reached a total release of 18% DNA (3.6 μg) at the end of day 3. Slow release was then observed from day 3 to 7, and about 0.5 μg DNA was released in these 4 days. These data indicated that PECE hydrogel might be a promising vector for sustained gene release.

### Distribution of genes inside PECE hydrogel

**Figure 12:**
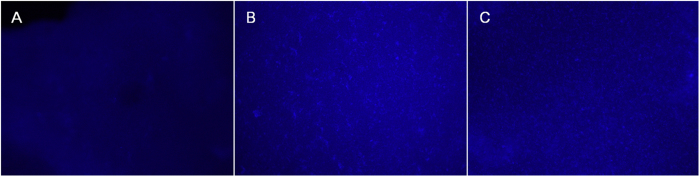
Distribution of gene inside PECE hydrogel stained with DAPI. (**A**) DNA, (**B**) PEA/DNA complexes, (**C**) FA-PEA/DNA complexes, the gene were stained with DAPI, and the pictures were taken with fluorescence inversion microscopy, ×50.

Gene complexes in aqueous solution were easy to aggregate due to the random motion that led to frequent collisions^34^,^35^. However, PEA/DNA and FA-PEA/DNA complexes had hardly any aggregation when dispersed into PECE hydrogel (Fig. 12). This can be attributed to the high viscosity of PECE hydrogel that slowed down the particle motion and the interaction between particles. The morphology of the gene particles was thus maintained and therefore, it became possible to deliver these gene complexes into targeted tumor cells.

### Transfection *in vitro* mediated by PECE hydrogel

**Figure 13:**
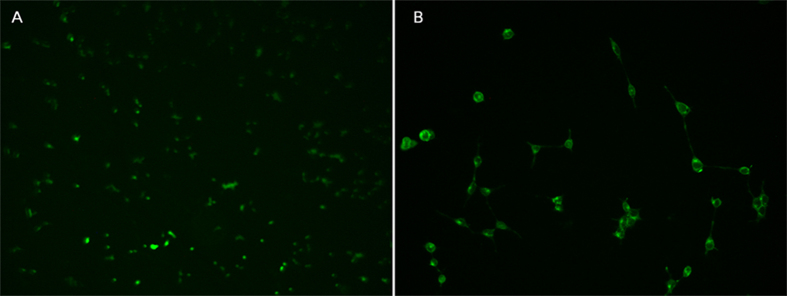
Transfection mediated with PECE hydrogel *in vitro*. Fluorescence inversion microscopy images of the 293T cell lines by the transfection of FA-PEA/eGFP (weight ratio was 2) polyplexes inside PECE hydrogel. (**A**) ×50, (**B**) ×200.

The genes inside the FA-PEA/DNA/PECE hydrogel could be delivered into cells after the release of FA-PEA/DNA complexes from PECE hydrogel. To test this hypothesis, FA-PEA/eGFP/PECE hydrogel was test with 293T cell lines. Fluorescence inversion microscopy was used to investigate the transfection efficiency qualitatively as shown in Fig. 13. It was found that eGFP expressed at a relative low level in the 293T cells, and this might be due to the small amount of gene in the release medium. The above results demonstrated that PECE hydrogel might be a potential gene delivery vector for sustained release of genes into cells.

## Discussion

In this study, we had synthesized a biodegradable tumor-targeted folate-poly (esteranmine) (FA-PEA) polymer based on low-molecular-weight polyethyleneimine, which was a favorable gene vector. The 1H-NMR spectrum indicated the successful synthesis of both PEA and FA-PEA polymers, which was then demonstrated to be biocompatible through MMT assay. The agarose gel electrophoresis was carried out and the results indicated DNA was successfully condensed into the complexes. The maximal transfection efficiency of both PEA and FA-PEA were demonsntrated to be higher than 40% in the C26 cells. Although the transfection efficiency of these polymers was slightly lower than that of PEI 25k, their cytotoxicity was much lower, thus these polyethyleneimine derivatives might be more favorable gene vectors than PEI 25k.

Wnt inhibitory factor-1 (WIF-1) was a secreted antagonist of Wnt signaling, and down regulation of WIF-1 had been reported in many kinds of cancers, such as non-small cell lung cancer^36^, colon cancer^37^ and so on. In this work, WIF-1 gene, as an anti-oncogene, was delivered into colon cancers with the help of the FA-PEA polymer. It was surprisingly found that the cell uptake of FA-PEA/WIF-1 complexes was much easier than that of raw WIF-1 genes, and FA-PEA/WIF-1 complexes could induce apoptosis at a very high level.

Moreover, we constructed a thermo-sensitive FA-PEA/DNA/PECE hydrogel system for the sustained release of genes complexes and successfully delivered the genes into tumor cells. The gene complexes were homodisperse and no aggregation was found in the hydrogel system. The thermo-sensitive FA-PEA/DNA/PECE hydrogel system showed great potential in targeted gene therapy and achieved local sustained release of antitumor genes.

## Materials and Methods

### Materials

Branched PEI (molecular weight 1800Da and 25kDa) were obtained from Alfa Aesar, USA. Isophorone di-isocyanate (IPDI), L-lactide, folic acid (FA), catalyst stannous octoate (Sn(Oct)2, 95%), 1-Ethyl-3-(3-dimethylaminopropyl) carbodiimide (EDC), N-Hydroxysuccinimide (NHS), 4′,6-Diamidino-2-phenylindole dihydrochloride (DAPI), Hoechst 33258, propidium iodide (PI), Dulbecoo’s Modified Eagle’s Medium (DMEM), Roswell Park Memorial Institute (RPMI)-1640 Medium (1640) and 3-(4,5-dimethylthiazol-2-yl)-2,5-diphenyltetrazolium bromide (MTT) were purchased from Sigma, USA. PECE hydrogel (PEG550-PCL2200-PEG550) was supplied by our group. Plasmid eGFP (green fluorescent protein) was offered by our group and after amplification was isolated and purified using a Qiagen plasmid purification kit following the protocol provided by manufacturer. Murine anti-oncogene WIF-1 (NM_011915.2) transformed into E.Coli JM109 was purchased as a sample from Biowit Technologies, China and after amplification was isolated and purified using a Qiagen plasmid purification kit. An Annexin V-FITC Apoptosis Detection Kit was supplied by Keygene biotech, China. Other chemicals were got from Chengdu KeLong Chemicals, China. They were all analytical pure grade and used as received.

### Cell lines and cell culture

C26 cell lines were incubated in Roswell Park Memorial Institute (RPMI)-1640 Medium (1640) containing 10% bovine serum and 1% antibiotics (penicillin-streptomycin, 100U/ML) at 37 °C in a humidified atmosphere containing 5% CO_2_. 293T cell lines were incubated in Dulbecoo’s Modified Eagle’s Medium (DMEM) containing 10% fetal bovine serum and 1% antibiotics (penicillin-streptomycin, 100 U/ML) at 37 °C in a humidified atmosphere containing 5% CO_2_. The cell lines in this paper were obtained from the American Type Culture Collection (ATCC).

### Synthesis and characterization of PEA and FA-PEA

Synthesis of PEA was conducted according to the method reported by our team previously^5^. First, we obtained poly (L-lactide) (PLLA) by ring-opening polymerization of L-lactide initiated by 1,4-butanediol, then NCO-ended PLLA polymer was obtained according to the reaction of isophorone di-isocyanate (IPDI) and PLLA. Finally we got poly (ester amine) (PEA) polymer by the reaction of NCO- on IPDI-PLLA-IPDI and NH_2_ on low molecular weight PEI 1800.

FA-PEA was synthesized by the reported method^25^ with some modification. Firstly, folic acid (FA) (0.5 mM) and PEA (0.5 mM) were respectively dissolved in PBS (pH 7.4). Then, 1-Ethyl-3-(3-dimethylaminopropyl) carbodiimide (EDC) (5 mM) and N-hydroxysuccinimide (NHS) (2.5 mM) were respectively added into the folic acid solution, and magnetically stirred for 30 minutes in order to activate the carboxyl of FA. Thereafter, the PEA solution was put into the FA mixture solution, and continued to be magnetically stirred for 24 hours. The mixture was purified by membrane dialysis (MWCO: 3500) in distilled water for 3 days and the solution was lyophilized. All of the experiments were conducted at room temperature and kept in a dark place. The chemical structure of PEA (in D_2_O) and FA-PEA (in D_2_O) were characterized on ^1^H Nuclear Magnetic Resonance (^1^H- NMR, 400MHZ, Varian, US).

### Preparation of polymer/DNA complexes

A plasmid DNA solution (0.5μg/ml) was prepared in TNE solution. Polymer (PEA or FA-PEA) was dissolved in distilled water to form a solution of 0.5 μg/ml, and DNA solution was added to the polymer solution at the desired weight ratio and mixed gently. The complexes were incubated at room temperature for 30 minutes before further application and characterization.

### Cell cytotoxicity assay

Cytotoxicity evaluation of polymers compared with PEI 25k was measured with an MTT (Sigma, USA) assay using C26 cell lines according to the manufacturer’s instructions. Cells were plated at a density of 4000 cells per well in 100 μl 1640 growth medium in 96-well plates and grown for 24 hr. The cells were then exposed to PEA, FA-PEA and PEI 25k at different concentrations for 24 hr, followed by the addition of 20 μl 3-(4,5-dimethylthiazol-2-yl)-2,5-diphenyltetrazoliumbromide (MTT) solution (5 mg/ml). After further incubation of 4 hours, the MTT solution was removed from each well, and 150 μl DMSO was added to dissolve the formazan crystals. The absorbance was recorded at 570 nm by an ELISA microplate reader (Bio-Rad). Polymer-untreated cells were used as a control.

### Gel retardation assay

The extension and efficiency of DNA condensation by polymers was conducted with a gel retardation assay. The polymer and plasmid DNA were diluted to a certain concentration with distilled water, and the plasmid DNA solution was added to the cationic polymer solution with the same volume at various polymer/DNA weight ratios and then mixed well. After incubation for 30 mins at room temperature, the polymer/DNA complexes formed. 10 μl of the solution was put on a 1% (w/v) agarose gel in a 1 × tris-acetic acid-EDTA (TAE) buffer at 120V for 30 min. The DNA bands stained with ethidium bromide were illuminated by ultraviolet Tran illumination and photographed with a Lumi-Imager.

### Preparation of hydrogel loaded with gene

FA-PEA/DNA complex was added into the PECE solution with the final PECE concentration of 21 Wt% to get a thermosensitive hydrogel composite system containing the gene. These samples were kept at 4 °C before use. The pictures of hydrogel in room temperature and 4 °C were taken by Nikon digital camera.

### Transfection efficiency *in vitro*

Efficiency of the developed cationic polymers to induce gene expression in C26 cells was determined using eGFP (green fluorescent protein) plasmids according with the standard protocol recommended by DharmaFECT, ThermoFisher Scientific, the optimal weight ratio of PEI/DNA was 1.5 in this paper without further statement.

In order to quantitatively evaluate the transfection efficiency of the two PEI derivatives *in vitro*, 2 μg of eGFP plasmid was respectively encapsulated with PEA and FA-PEA at different ratios to transfect into C26 cells. Cells were plated at a density of 2 × 10^5^ cells per well in 2 ml 1640 growth medium in 6-well plates and grown overnight, then the culture medium was replaced with serum free medium containing polymer/DNA complexes. After incubation for 4 hr, the culture media was removed, and the cells were washed with 1640 growth medium and replaced by fresh growth medium. After 24 hr, the culture medium was removed, and the cells were collected to detect the transfection efficiency using flow cytometry. Furthermore, the transfection images were observed by a DM2500 fluorescence inverted microscope (Leica Microsystems CMS GmbH, Wetzlar, Germany) and photographed by Spot Flex in order to qualitatively analyze the transfection efficiency.

### Apoptosis detections *in vitro*

The apoptosis of C26 cell lines *in vitro* by the treatments of complexes contained murine tumor suppressor gene WIF-1 was quantitively detected by Annexin V-FITC Apoptosis Detection Kit (KeyGEN BioTECH, China) followed by the manufacturers’ experimental protocols. C26 cells were plated at a density of 2 × 10^5^ cells per well in 2 ml 1640 growth medium in 6-well plates and incubated for the night, then PEA/WIF-1 and FA-PEA/WIF-1 complexes were delivered into C26 cell lines and incubated for 4 hours with serum free medium, the culture media was removed, the cells were washed with 1640 growth medium and replaced by fresh growth medium. PEA and FA-PEA were treated in the same way as control, the concentration of WIF-1 was 2 μg/cell, PEA and FA-PEA were all 4 μg/cell. Finally the cells were collected and stained with Annexin V-FITC Apoptosis Detection Kit, then detected with flow cytometry.

Meanwhile, the cells were also stained with PI/Hoechst 33258 and DAPI respectively according to the manufacturers’ protocols, and the apoptotic bodies were observed by DM2500 fluorescence inverted microscope (Leica Microsystems CMS GmbH, Wetzlar, Germany), the images were obtained by Spot Flex in order to qualitatively analyze the cell apoptosis, according to the manufacturer’s protocol.

### Sustained release of gene from PECE hydrogel

The prepared PEA/DNA, FA-PEA/DNA complexes and DNA loaded PECE solutions (the PECE concentration was 21% and the gene was 20 μg) were incubated at 37 °C for 12 hr, and 4 ml PBS 7.4 solution were added. Then at given times (t = 0.5 hr, 1 hr, 2 hr, 4 hr, 6 hr, 8 hr, 12 hr, 24 hr, 48 hr, 72 hr, 96 hr) 1 ml release medium was replaced by 1 ml fresh PBS 7.4 solution. The genes in release media were stained with Hoechst 33258 and detected with LS55 fluorescence spectrophotometer (Perkin-Elmer Limited Co.) according to the method reported by Penketh, P. G. *et al.*^38^.

### Distribution of genes inside PECE hydrogel

The prepared PECE solution loaded PEA/DNA, FA-PEA/DNA complexes and DNA (the PECE concentration was 21% and the gene was 5 μg) was exposed at 37 °C to form hydrogel, then the hydrogel stained with DAPI working solution for 10 min before imaging with DM2500 fluorescence inverted microscope (Leica Microsystems CMS GmbH, Wetzlar, Germany) to show the distribution of genes inside the hydrogel following the method reported by Lei, Y. *et al.*^3^.

### Transfection *in vitro* mediated by PECE hydrogel

The prepared FA-PEA/eGFP complexes loaded PECE solution (the PECE concentration was 21% and the gene was 20 μg) was put into 6-well plates and incubated at 37 °C for 12 hr to completely form hydrogel, 2 ml DMEM serum free medium was added, and the release medium was obtained after incubating for 48 hr. 293T cells were plated at a density of 2 × 10^5^ cells per well in 2 ml DMEM growth medium in 6-well plates and grown overnight, then the culture medium was replaced with the release medium. After incubation for 4 hr, the culture medium was removed, and the cells were washed with DMEM growth medium and replaced by fresh growth medium. After 24 hr, the culture medium was removed, and the cells were observed by DM2500 fluorescence inverted microscope (Leica Microsystems CMS GmbH, Wetzlar, Germany) and the images were obtained by Spot Flex in order to qualitatively analyze the transfection efficiency. This experiment was conducted according to the above experimental protocol.

### Statistical Analysis

All data expressed as mean ± standard deviation were representative of at least three independent experiments. Data were statistically evaluated using one-way analysis of variance (ANOVA) test.

## Additional Information

**How to cite this article**: Yang, Y. *et al.* A novel gene delivery composite system based on biodegradable folate-poly (ester amine) polymer and thermosensitive hydrogel for sustained gene release. *Sci. Rep.* **6**, 21402; doi: 10.1038/srep21402 (2016).
